# Safety of Allogeneic Umbilical Cord Blood Stem Cells Therapy in Patients with Severe Cerebral Palsy: A Retrospective Study

**DOI:** 10.1155/2015/325652

**Published:** 2015-07-08

**Authors:** Mei Feng, Aili Lu, Hongxia Gao, Caiwen Qian, Jun Zhang, Tongxiang Lin, Yuanqi Zhao

**Affiliations:** ^1^Department of General Internal Medicine, Guangdong Provincial Hospital of Chinese Medicine, Guangzhou 510120, China; ^2^The School of Medicine, Jinan University, Guangzhou 510632, China; ^3^Shenzhen Beike Cell Engineering Research Institute, Shenzhen 518057, China

## Abstract

This retrospective study aimed to assess the safety of patients with severe cerebral palsy (CP), who received allogeneic umbilical cord blood stem cells (UCBSCs) treatment from August 2009 to December 2012 in Guangdong Provincial Hospital of Chinese Medicine. A total of 47 patients with average age of 5.85 ± 6.12 years were evaluated in this study. There was no significant association with allogeneic UCBSCs treatments found in the data of the laboratory index . No casualties occurred. Some adverse events during treatments were found in 26 (55.3%) patients, including fever (42.6%) and vomiting (21.2%). Intrathecal infusion and the ages at the initiation of treatment (≤10 years old) were risk factors for the occurrence of adverse events by logistic regression analysis. However, all adverse events disappeared after symptomatic treatment. No treatment related serious adverse events were found in follow-up visits within 6 months. In conclusion, allogeneic UCBSCs treatment was relatively safe for severe CP patients.

## 1. Introduction

Cerebral palsy (CP) is a group of permanent disorders of the development of movement and posture, causing activity limitations attributed to nonprogressive disturbance that occur in the developing fetal or infant brain. The motor disorders of cerebral palsy are often accompanied by disturbances of sensation, perception, cognition, communication, and behavior, by epilepsy and by secondary musculoskeletal problems [1]. As one of the most common causes of physical disability in childhood, CP has an approximate prevalence of 1.5–3.6 per 1,000 individuals [2, 3]. Despite extensive treatment, neurological impairments still eventually lead CP patients to lifelong disability [4]. Furthermore, while traditional treatment can only bring improvement for sufferers of mild to moderate CP, severe CP lacks effective intervention options [5].

During the past two decades, many researchers have assessed the efficacy and safety of cell therapy for replacing lost cells and reducing brain damage [6]. Among stem cell sources, umbilical cord blood (UCB) is currently a popular source of adult stem cells which contains stem cells with variable therapeutic potential [7]. UCB cells are not only immune naive with little serious rejection, but also able to differentiate into other phenotypes, including the neural lineage [8, 9]. Thus, UCBSCs could potentially be effective in treating children with CP.

Several preclinic and clinic trials now show that allogeneic UCBSCs have therapeutic effects for CP [10, 11] and low immunogenicity, noninvasive collection, few ethical issues, and “off-the-shelf” source are their advantages [12, 13]. However, its usage is still limited with the safety and efficacy concerns in clinical trials. The risks of stem cell therapy occur primarily with allogeneic transplants, which expose the recipient to graft-versus-host disease (GVHD). While most reports of complications were in children undergoing hematopoietic stem cell (HSC) transplantation for malignancies, there has been no systematic report on complication from non-HSC transplantation [14]. We are wondering if allogeneic UCBSCs are safe for treatment of patients with CP. A protocol of allogeneic UCBSCs infusion treating cerebral palsy had been established in the earlier stage and the preliminary evaluation on the effectiveness of the treatment for severe cerebral palsy had been carried on in our hospital [15]. Thus, this retrospective study was conducted to assess the safety of allogeneic UCBSCs treatment in patients with severe CP.

## 2. Patient and Method

### 2.1. Study Design and Population

This study included 47 patients with severe CP who received allogeneic UCBSCs treatment from August 2009 to December 2012 at the Guangdong Provincial Hospital of Chinese Medicine. Data were obtained from medical records in an electronic database of our hospital. Among those cases, patients who met the following inclusion criteria were selected: (1) diagnosis of cerebral palsy [1]; (2) severe CP symptoms after a formal rehabilitation therapy over six months without amelioration, which were grade IV or grade V referring to gross motor function classification system (GMFCS) [16]; (3) no active cardiac, pulmonary, renal, or hepatic gastrointestinal disease; (4) receiving a course of UCBSCs treatment and complete safety detective indicators before and after treatment. Patients were excluded if they had a previous history of severe allergic reactions or dropped out before 6 months of follow-up.

Of the 62 patients who underwent allogeneic UCBSCs treatment during the study period, 9 patients did not meet the inclusion/exclusion criteria, 2 lacked complete medical records, and 4 were missing follow-up. Thus, the study consisted of 47 patients. Peripheral blood samples were collected two days before the first treatment and one day after the last treatment. Clinical parameters were collected from admission to 6 months after treatment.

### 2.2. Cell Infusion and Patient Monitoring

With doses of 2-3 × 10^7^ cells per injection, 4–8 injections that depended on health conditions were performed for each patient. In detail, we performed cell injection 4 times for a single patient. In order to provide patients maximal benefits from this treatment, we might increase more injection times, up to 8 times if the patients had no adverse events in the treatment period. The first injection was intravenous infusion and the rest were intrathecal injections. The intervals between injections were 3–5 days. If any reactions, such as fever, acute respiratory infections, and epilepsy, were observed at any time during transplantation treatment, UCBSCs injection was not performed until recovery.

### 2.3. Data Collections

The following clinical data were investigated from patients: clinical characters of patients, laboratory indexes before and after treatment, way of allogeneic UCBSCs treatment (intrathecal or intravenous), type of adverse events, time of adverse events occurrence, time of adverse events remission, method of symptomatic therapy, and characteristics of birth which included birth method, birth weight, gestational age, neonatal jaundice (yes or no), neonatal hypoxic ischemic encephalopathy (yes or no), epilepsy (yes or no), and mother infected during pregnancy (yes or no).

#### 2.3.1. Laboratory Indexes

Collection items of laboratory indexes pre- and posttreatment included leukocytes, erythrocytes, hemoglobin, platelets, alanine aminotransferase (ALT), aspartate aminotransferase (AST), serum urea nitrogen (BUN), serum creatinine (SCR), serum potassium (K^+^), serum sodium (Na^+^), serum chloride ion (CL^−^), total carbon dioxide (TCO_2_), serum glucose (Glu), prothrombin time (PT), activated partial thromboplastin time (APTT), international normalized ratio (INR), and fibrinogen (FIB). In addition, HIV, syphilis rapid plasma reagin (RPR), and hepatitis markers were also assessed.

#### 2.3.2. Adverse Events Reporting and Follow-Up

Adverse events which occurred among patients with allogeneic UCBSCs treatment in hospital were carefully observed and recorded in detail by the clinicians who provided care. Parents or caregivers were also instructed on how to identify signs and symptoms of adverse events before the treatment. At the same time, they were also requested to report whatever untoward event they observed following the infusion, whether or not they believed that to be related to this treatment. Patients could receive the necessary medical assistance provided by medical staff. A follow-up was carried out with each patient or his parent at the first, second, and sixth month through telephone or email after discharge, with adverse events reporting from their parents or caregivers.

All study participants or their guardians provided written informed consent on receiving allogeneic UCBSCs treatment and the study protocol was approved by the Institutional Review Board at the Guangdong Provincial Hospital of Chinese Medicine.

### 2.4. Statistical Analysis

Descriptive analysis was performed to describe the baseline characteristics of patients included and the frequency of adverse events after UCBSCs treatment. Comparisons between continuous variables were based on a paired *t*-test if variables were normally distributed and on a Mann-Whitney *U* test when this was not the case. Frequencies of categorical variables were compared with chi-square or Fisher's exact test. In order to find out the association between the occurrence of an adverse event during allogeneic UCBSCs treatment and several potential risk factors, logistic regression was performed. Statistical analyses were performed using SPSS 17.0 (SPSS Inc., Chicago, IL, USA).

## 3. Results

### 3.1. Demography

The demographic characteristics of all patients included in this study are summarized in Table 1. Of the 47 patients, 35 (74.5%) were males and 12 (25.5%) were females. The age of the patients was 5.85 ± 6.12 (mean ± SD) years, which ranged from 1 to 29 years, including 12 patients at ages of over 10 years. A course of treatment consisted of 4–8 times UCBSCs infusions (mean ± SD: 5.38 ± 1.36 times) and the mean duration of treatment was 27.17 ± 8.03 days.

### 3.2. Laboratory Tests

No significant difference was found in leukocytes, erythrocyte, hemoglobin, platelets, ALT, AST, BUN, SCR, K^+^, Na^+^, CL^−^, Glu, TCO_2_, or blood coagulation parameters (PT, APTT, AT, INR, and FIB), both before and after UCBSCs treatment (*P* > 0.05) (Table 2). Hepatitis markers (HAV, HBV, HCV, HDV, and HEV), HIV, and RPR tests were all negative after the treatment.

### 3.3. Adverse Events

Analysis of frequency of adverse events after allogeneic UCBSCs infusions indicated that there were some nonfatal adverse events. There was no adverse event in the initial intravenous infusion. All of the adverse events occurred during the rest period when the injection was intrathecal infusion. 26 out of 47 patients (55.3%) had reported adverse events, but no casualties occurred. Fever and vomiting were the most common adverse events, with incidences of 42.6% and 21.2%, respectively. There were 3 (6.4%) cases of seizures and 3 (6.4%) cases of headaches for each. Two (4.3%) upper respiratory tract infections and 2 (4.3%) episodes of dermatitis occurred in the group. Only 1 (2.1%) case of waist pain and constipation occurred. No diarrhea, insomnia, pneumonia, or anorexia occurred in this group of patients. All adverse events disappear after symptomatic treatment, with the remission time ranging from 8 to 72 hours (Table 3).

### 3.4. Risk Factors for the Occurrence of an Adverse Event

In order to reveal other potential risk factors associated with the occurrence of an adverse event during the treatment, we fitted a logistic regression model. Factors tested included gender (male or female), birth method (vaginal or cesarean), birth weight (normal or low), gestational age (preterm or full term), neonatal jaundice (yes or no), neonatal hypoxic ischemic encephalopathy (yes or no), epilepsy (yes or no), mother infected during pregnancy (yes or no), and age at treatment initiation (>10 years old or ≤10 years old). Before performing the logistic regression, we conducted correlate analysis to test whether there existed significant associations among independent variables. We found no colinearity and therefore proceeded to perform the logistic regression. According to the binary logistic regression analysis, the significant risk factor was age at treatment initiation (≤10 years old) (*P* = 0.036, OR = 12.543, 95% CI: 1.178–133.549). However, other variables did not appear to influence the occurrence of adverse events (Table 4).

### 3.5. Follow-Up

Follow-up with each patient was performed via telephone or email at the first, second, and sixth month after their discharge. While 15 adverse events, including 7 upper respiratory tract infection, 4 diarrhea, 3 fever, and 1 seizure, happened in patients among all the 47 patients during the sixth month follow-up, there was no evidence showing that these common diseases were associated with the cell transplantation treatments. No treatment related serious adverse events, such as death, GVHD, central nervous system infection, and pneumonia, appeared in any one of all patients.

## 4. Discussion

UCBSCs are rich in mesenchymal progenitor cells [17] and contain a large number of endothelial cell precursors [18]. They have been used clinically for over 20 years as a cell source for hematopoietic stem cell transplantation. Beyond this, cord blood and umbilical cord-derived stem cells have demonstrated potentials for pluripotent lineage differentiation including liver, pancreatic, and neural tissues, both in vitro and in vivo. This promising research has opened up a new era for the utilization of neonatal stem cells, now used beyond hematology in clinical trials for autoimmune disorders, cerebral palsy, or type I diabetes [19, 20]. A preclinical study found that the long-lasting positive influence of UCBCs is derived from their paracrine effects which stimulated recovery in the injured brain and protected against further brain damage in rat trials with CP [10]. Clinical studies have also shown whether autologous UCBCs or allogeneic UCBSCs have therapeutic benefits in patients with CP [11, 21].

Although autologous UCBSCs would be a better choice for the treatment of CP patients, but most children with CP haven't banked their own UCB before this treatment. In Australia, it was not feasible to conduct trials of autologous UCBSCs for children with CP until early 2013 because of relatively low per capita cord blood storage in CP patients [12], so allogeneic UCBSCs would be a good alternative treatment option. Interestingly, another CP clinical study showed that the allogeneic treatment group demonstrated significantly decreased levels of proinflammatory factors, such as interleukin- (IL-) 1*α*, interleukin- (IL-) 6, tumor necrosis factors- (TNF-) *β*, and RANTES, and showed a statistically significant improvement in motor and social behavior compared to the autologous treatment group [22]. This indicates that allogeneic stem cells may be more beneficial than autologous cells for CP patients. Our previous study also found that UCBC treatment could significantly improve gross motor function measure (GMFM) scores in patients with severe cerebral palsy, especially in supine, prone, and sitting functions [15]. The transplantation with UCBCs without full HLA matching still had lower incidences of GVHD, graft-versus-leukemia (GvL), and tumorigenicity as well as infectious complications in other circumstance reported by previous studies [23, 24]. In our clinical trial circumstance, allogeneic UCBSCs without full HLA matching in absence of immune suppression/myeloablation to treat nonhematopoietic conditions showed to be even safer than those mentioned before [25].

The results of the present study on allogeneic UCBSCs infusion in patients with severe CP who did not have immunosuppression or myeloablation suggest that this strategy could be a safe therapeutic approach. Compared with blood samples before treatment, there was no statistical significance among leukocytes, erythrocytes, hemoglobin, platelets, ALT, AST, BUN, SCR, K^+^, Na^+^, CL^−^, Glu, TCO_2_, or blood coagulation parameters (PT, APTT, AT, INR, and FIB). This is similar to previous studies [25, 26]. Hepatitis markers (HAV, HBV, HCV, HDV, and HEV), HIV, and RPR tests were all negative after the treatment. It demonstrates that the risk of communicable disease could be avoided if the cell processing test was conducted in a sterile fashion.

No serious adverse events (e.g., death, GVHD, central nervous system infection, and pneumonia) were observed during the period of treatment. Fever was the most common adverse event. These findings were consistent with studies that recorded the similar adverse event [11, 26]. This suggests that allogeneic UCBCs can still cause mild immunological rejection reactions in vivo [13]. As the second common adverse reaction, vomiting ([12.00 ± 3.35] h) had occurred earlier than fever ([19.91 ± 21.76] h). One reason was likely related to allogeneic UCBCs treatment, another reason might be the side effect of general anesthesia. Postoperative vomiting was the most common complication after general anesthesia among infants and children, and the incidence rate ranged from 8.9% to 42% among susceptible children [27]. Furthermore, 6 months of follow-up in this study showed that no treatment related serious adverse events were reported. Even though the time of follow-up was shorter than the study which performed for 1 year follow-up evaluation [11], both of them had no prolonged or delayed onset of serious adverse effects reported.

The manifestation of different incidences of the adverse events between intravenous infusion and intrathecal infusion perhaps implies that the way of allogeneic UCBSCs infusion associated with an adverse event. Moreover, logistic regression model analysis showed that age in the initiation of treatment (≤10 years old) was the risk factor for adverse events. So we deduce that intrathecal infusion and the age in the initiation of treatment (≤10 years old) might be associated with the occurrence of adverse events. This finding potentially demonstrates that the present single dosage (2-3 × 10^7^/dose) of allogeneic UCBSCs is too large for patients whose age in the initiation of treatment was less than or equal to 10-year-olds who received intrathecal injection in this study. Due to lack of a unified worldwide standard for the optimal cell number of UCBSCs treatment currently, this dosage was on the basis of the previous study [25] and the clinical experience of our exploratory stem cell treatments. In fact, a dose-response association could not be observed. This is the limitation of our study design. Another possible reason is that humoral immunity almost reached the level of adults when children were 10 years old. Therefore, improved immune system may decrease the occurrence of adverse events. So in order to reduce the adverse events, the future clinical study should be focused on how to calculate the cell numbers individually based on the weight or age of patients in CP. However, no definite conclusions can be drawn from small sample trials, and more studies are needed to further investigate and confirm these findings.

While traditional use of cord blood derived stem cells in the treatment of cerebral palsy has been restricted to the autologous setting, our approach has provided an “off-the-shelf” source of stem cells. However, some major concerns are still in debate. For example, the cell transplantation might induce GVHD due to HLA mismatch. Our study showed that only little mild reactions, such as fever or vomiting, happened in our clinical experiments without severe immune reactions observed. As we mentioned in the Introduction, the UCBCs are naïve cells, which might cause little severe immune rejection. Based on our research data, the cell transplantation is free from the serious rejection of GVHD with only mild reactions which could be recovered without special treatment in a couple of days. However, several other concerns, such as chronical risks and tumorigenicity, have not been answered in this study due to limited financial and other research resources. We need many other experiments to provide more efficient treatments and to avoid possible side effects in the future.

## 5. Conclusion

The trial of allogeneic UCBSCs therapy for patients with severe CP is relatively safe during treatment and in 6-month follow-up as we carried out. Adverse events during treatment were mild and could be recovered without special treatment. Logistic regression analysis showed that intrathecal infusion and ages at the initiation of treatment (≤10 years old) might be associated with the occurrence of the adverse events.

## Figures and Tables

**Table 1:** Characteristics of 47 patients with severe CP who were treated with allogeneic UCBSCs.

Variables	Values
Mean age (SD), y	5.85 (6.12)
Age ranges (<10 y/≥10 y)	35/12
Gender	
Male *n* (%)	35 (74.5)
Female *n* (%)	12 (25.5)
Duration of treatment (SD), d	27.17 (8.03)
Number of infusions (SD), *t*	5.38 (1.36)
Birth method	
Vaginal *n* (%)	28 (59.6)
Cesarean *n* (%)	19 (40.4)
Mean birth weight (SD), kg	2.53 (0.84)
Low birth weight (BW <3000 g) *n* (%)	20 (42.6)
Mean gestational age (SD), w	35.06 (5.18)
Neonatal jaundice *n* (%)	8 (17.0)
NHIE *n* (%)	28 (59.6)
Epilepsy *n* (%)	13 (27.7)
MIDP *n* (%)	16 (34.0)

*Note.* NHIE: neonatal hypoxic ischemic encephalopathy; MIDP: mother infected during pregnancy; SD: standard deviation; y: year; kg: kilogram; w: week; d: day; *t*: time.

**Table 2:** Analysis of blood test before and after allogeneic UCBSCs treatment.

Parameter	Before treatment (*n* = 47) *n* (%)^∗^	After treatment (*n* = 47) *n* (%)^∗^	*P* value (two sides)	Reference range
Leukocytes	8 (17.0%)	13 (27.7%)	0.322	(5.0~12.0) × 10^9^/L
Erythrocytes	3 (6.4%)	8 (17.0%)	0.198	(4.0~5.5) × 10^12^/L
Haemoglobin	5 (10.6%)	7 (14.9%)	0.759	(110.0~150.0) g/L
Platelets	29 (61.7%)	23 (48.9%)	0.300	(100.0~300.0) × 10^9^/L
ALT	3 (6.4%)	5 (10.6%)	0.714	(9~50.0) U/L
AST	6 (12.8%)	5 (10.6%)	1.000	(15~40.0) U/L
BUN	8 (17.0%)	6 (12.8%)	0.773	(1.43~6.78) mmol/L
SCR	2 (4.3%)	2 (4.3%)	1.000	(16.0~73.0) umol/L
K^+^	3 (6.4%)	5 (10.6%)	0.714	(3.5~5.3) mmol/L
Na^+^	6 (12.8%)	10 (21.3%)	0.206	(137.0~147.0) mmol/L
CL^−^	4 (8.5%)	5 (10.6%)	1.000	(99.0~110.0) mmol/L
TCO_2_	16 (34.0%)	13 (27.7%)	0.656	(20.0~28.0) mmol/L
Glu	3 (6.4%)	2 (4.3%)	1.000	(3.9~6.1) mmol/L
PT	4 (8.5%)	4 (8.5%)	1.000	(10.0~13.0) s
APTT	11 (23.4%)	10 (21.3%)	1.000	(22.0~32.0) s
INR	1 (2.1%)	3 (6.4%)	0.308	0.8–1.2
FIB	5 (10.6%)	8 (17.0%)	0.552	(2.0~4.0) g/L

^∗^ The numbers (percentage) of patients whose blood test result was abnormal; UCBSCs: umbilical cord blood stem cells; ALT: alanine aminotransferase; AST: aspartate aminotransferase; BUN: serum urea nitrogen; SCR: serum creatinine; K^+^: serum potassium; Na^+^: serum sodium; CL^−^: serum chloride ion; TCO_2_: total carbon dioxide; Glu: serum glucose; PT: prothrombin time; APTT: activated partial thromboplastin time; INR: international normalized ratio; FIB: fibrinogen.

**Table 3:** Frequency of adverse events after allogeneic UCBSCs treatment.

Type of adverse events	Number of reactions (%)	Time of occurrence (h)	Time of remission (h)
Fever	20 (42.6%)	19.91 ± 21.76	28.62 ± 17.38
Vomiting	10 (21.2%)	12.00 ± 3.35	20.00 ± 6.20
Seizure	3 (6.4%)	19.50 ± 11.90	21.00 ± 6.00
Headache	3 (6.4%)	11.50 ± 7.78	36.00 ± 16.97
Upper respiratory tract infection	2 (4.3%)	38.50 ± 40.34	24.00 ± 0.00
Dermatitis	2 (4.3%)	12.50 ± 0.71	24.96 ± 13.34
Waist pain	1 (2.1%)	8.00	24.00
Constipation	1 (2.1%)	24.00	12.00
Diarrhea	0 (0%)	—	—
Insomnia	0 (0%)	—	—
Pneumonia	0 (0%)	—	—
Anorexia	0 (0%)	—	—

*Note*. UCBSCs: umbilical cord blood stem cells; h: hour.

**Table 4:** Logistic regression analysis for the association between the occurrence of adverse event during allogeneic UCBSCs treatment and several predicting factors.

Predicting factors	Significance	Odds ratio	95% confidence interval	
			Lower bound	Upper bound
Sex				
Female	0.124	4.420	0.664	29.417
Male	—	—	—	—
Birth method				
Cesarean	0.381	0.500	0.106	2.362
Natural birth	—	—	—	—
Birth weight				
Low birth weight	0.503	0.523	0.078	3.484
Normal birth weight	—	—	—	—
Gestational age				
Preterm	0.145	4.494	0.595	33.961
Full term	—	—	—	—
Neonatal jaundice				
Yes	0.796	1.287	0.191	8.669
No	—	—	—	—
NHIE				
Yes	0.599	0.681	0.163	2.847
No	—	—	—	—
Epilepsy				
Yes	0.627	1.456	0.320	6.623
No	—	—	—	—
MIDP				
Yes	0.605	1.514	0.314	7.289
No	—	—	—	—
Age at treatment initiation				
≤10 years old	0.036	12.543	1.178	133.549
>10 years old	—	—	—	—

*Note.* UCBSCs: umbilical cord blood stem cells; NHIE: neonatal hypoxic ischemic encephalopathy; MIDP: mother infected during pregnancy.

## References

[1] Rosenbaum P., Paneth N., Leviton A., Goldstein M., Bax M. (2007). A report: the definition and classification of cerebral palsy April 2006. *Developmental Medicine and Child Neurology. Supplement*.

[2] Blair E., Watson L. (2006). Epidemiology of cerebral palsy. *Seminars in Fetal and Neonatal Medicine*.

[3] El-Tallawy H., Farghaly W., Shehata G. (2014). Cerebral palsy in Al-Quseir City, Egypt: prevalence, subtypes, and risk factors. *Neuropsychiatric Disease and Treatment*.

[4] Patel D. R., Greydanus D. E., Calles J. L., Pratt H. D. (2010). Developmental disabilities across the lifespan. *Disease-a-Month*.

[5] Luan Z., Liu W., Qu S. (2012). Effects of neural progenitor cell transplantation in children with severe cerebral palsy. *Cell Transplantation*.

[6] Moon J.-H., Kim M. J., Song S.-Y. (2013). Safety and efficacy of G-CSF mobilization and collection of autologous peripheral blood stem cells in children with cerebral palsy. *Transfusion and Apheresis Science*.

[7] Lee M. W., Jang I. K., Yoo K. H., Sung K. W., Koo H. H. (2010). Stem and progenitor cells in human umbilical cord blood. *International Journal of Hematology*.

[8] Park D.-H., Lee J.-H., Borlongan C. V., Sanberg P. R., Chung Y.-G., Cho T.-H. (2011). Transplantation of umbilical cord blood stem cells for treating spinal cord injury. *Stem Cell Reviews and Reports*.

[9] Park D.-H., Borlongan C. V., Willing A. E. (2009). Human umbilical cord blood cell grafts for brain ischemia. *Cell Transplantation*.

[10] Bae S. H., Kong T. H., Lee H. S. (2012). Long-lasting paracrine effects of human cord blood cells on damaged neocortex in an animal model of cerebral palsy. *Cell Transplantation*.

[11] Min K., Song J., Kang J. Y. (2013). Umbilical cord blood therapy potentiated with erythropoietin for children with cerebral palsy: a double-blind, randomized, placebo-controlled trial. *Stem Cells*.

[12] Crompton K. E., Elwood N., Kirkland M., Clark P., Novak I., Reddihough D. (2014). Feasibility of trialling cord blood stem cell treatments for cerebral palsy in Australia. *Journal of Paediatrics and Child Health*.

[13] Lee M., Jeong S. Y., Ha J. (2014). Low immunogenicity of allogeneic human umbilical cord blood-derived mesenchymal stem cells *in vitro* and *in vivo*. *Biochemical and Biophysical Research Communications*.

[14] Carroll J. E., Mays R. W. (2011). Update on stem cell therapy for cerebral palsy. *Expert Opinion on Biological Therapy*.

[15] Feng M., Gao H. X., Dai X. P. (2011). Clinical observation on the efficacy of umbilical blood stem cells transplantation in 30 cases with severe cerebral palsy. *Chinese Journal of Blood Transfusion*.

[16] Palisano R., Rosenbaum P., Walter S., Russell D., Wood E., Galuppi B. (1997). Development and reliability of a system to classify gross motor function in children with cerebral palsy. *Developmental Medicine and Child Neurology*.

[17] Erices A., Conget P., Minguell J. J. (2000). Mesenchymal progenitor cells in human umbilical cord blood. *British Journal of Haematology*.

[18] Nieda M., Nicol A., Denning-Kendall P., Sweetenham J., Bradley B., Hows J. (1997). Endothelial cell precursors are normal components of human umbilical cord blood. *British Journal of Haematology*.

[19] Forraz N., Mcguckin C. P. (2011). The umbilical cord: a rich and ethical stem cell source to advance regenerative medicine. *Cell Proliferation*.

[20] McGuckin C. P., Forraz N., Allouard Q., Pettengell R. (2004). Umbilical cord blood stem cells can expand hematopoietic and neuroglial progenitors in vitro. *Experimental Cell Research*.

[21] Jensen A., Hamelmann E. (2013). First autologous cell therapy of cerebral palsy caused by hypoxic-ischemic brain damage in a child after cardiac arrest—individual treatment with cord blood. *Case Reports in Transplantation*.

[22] Bae S. H., Lee H. S., Kang M. S., Strupp B. J., Chopp M., Moon J. (2012). The levels of pro-inflammatory factors are significantly decreased in cerebral palsy patients following an allogeneic umbilical cord blood cell transplant. *International Journal of Stem Cells*.

[23] Kurtzberg J. (2009). Update on umbilical cord blood transplantation. *Current Opinion in Pediatrics*.

[24] Reddi A. S., Kuppasani K., Ende N. (2010). Human umbilical cord blood as an emerging stem cell therapy for diabetes mellitus. *Current Stem Cell Research and Therapy*.

[25] Yang W.-Z., Zhang Y., Wu F. (2010). Safety evaluation of allogeneic umbilical cord blood mononuclear cell therapy for degenerative conditions. *Journal of Translational Medicine*.

[26] Zhang L. X., Xing L. H., Zhang L. L. (2010). Clinical safety of umbilical cord blood stem cells for treatment of children with cerebral palsy. *Chinese General Practice*.

[27] Kovac A. L. (2007). Management of postoperative nausea and vomiting in children. *Pediatric Drugs*.

